# Iodine Bath Treatment of Empyema

**Published:** 1905-03-11

**Authors:** 


### Hospital Clinics.

### IODINE BATH TREATMENT OF EMPYEMA.

The rapidity with which cases of empyema progress to cure is very variable even after apparently successful operations for their drainage. This variation depends upon a number of factors, principally the nature of the causative organism and the duration of the effusion. At all periods of life a majority, and in childhood a very large majority, of empyemata are due to the pneumococcus; and in the case of a pneumococcal empyema of the ordinary type—that is to say, an abscess containing thick creamy pus—the interval between drainage and closure of the wound is not often more than a week or two, unless the pus has been in the pleural cavity for a long time. In the latter case the prolonged compression of the lung will naturally delay its expansion when the fluid is removed. But even when expansion is delayed it is unusual for an uncomplicated pneumococcal empyema to become fœtid after drainage, or for symptoms to persist after operation unless a pocket of pus is overlooked or the drainage is insufficiently free. But occasionally, for inexplicable reasons, the reaction of the walls of the cavity is reduced, and then may be found a sinus continuing to discharge pus, for long periods, while constitutional symptoms persist. These cases are always anxious, for even though the pneumococcus may have been recovered from the pus, it is impossible to say with assurance that there is not a pulmonary tuberculosis underlying the pleural lesion. For these cases Dr. Langdon Brown[1] recommends iodine baths. The wisdom of irrigating the pleural cavity is variously estimated, and there appears little doubt that occasionally accidents in the nature of syncope have attended the practice. The common method of carrying out the procedure has involved the use of a funnel, the solution being run in by gravity. Dr. Brown suggests the immersion of the patient in an iodine bath. The strength of the bath is two drachms of liquor iodi fortis to the gallon of water. In this bath the patient is placed for a quarter of an hour once daily. By this means the inflow and return of the irrigating medium are automatically controlled by the respiratory movements.

The recommendations of this treatment, according to the author, are as follows:—1. There is no possibility of the fluid being forced into the pleural cavity under pressure, since the respiratory movements themselves form the pump which draws the fluid in. 2. The free dilution of the pus in the cavity greatly diminishes septic absorption, as is seen in the improvement in the temperature and constitutional condition. 3. Any fœtor in the pus is speedily diminished, and usually disappears entirely. It is to these cases of "stinking empyema" that the treatment is specially applicable. 4. It is entirely free from danger and hastens convalescence.

Dr. Brown cites a number of cases in which the iodine bath treatment seemed to be beneficial, and the first of the series in particular is sufficiently convincing to be recorded. A lad of 18, two months before admission, had been operated on for the drainage of an empyema in the left side. Soon after his discharge he had a recurrence of pain in that side, and on examination presented a red, glazed, fluctuating scar, with signs of fluid at the corresponding base of the chest. The scar gave way and offensive pus was discharged, and also, it may be said, expectorated. Five days later his temperature was still hectic, and the discharge very offensive, while his cough was incessant. Iodine baths were begun, with immediately good results. The pus lost its fœtor, the discharge rapidly diminished, the cough ceased, and the temperature never again rose above the normal.

It would seem that we have in this treatment a valuable instrument for the cure of a distressing and tedious complication of empyema.

[1] St. Bart's Hospital Jour., March 1905.

